# Decreased expression of the long non-coding RNA SLC7A11-AS1 predicts poor prognosis and promotes tumor growth in gastric cancer

**DOI:** 10.18632/oncotarget.22486

**Published:** 2017-11-07

**Authors:** Yajun Luo, Cheng Wang, Peng Yong, Pengcheng Ye, Zilin Liu, Zhiming Fu, Fei Lu, Wanping Xiang, Wang Tan, Jiangwei Xiao

**Affiliations:** ^1^ The Department of Gastrointestinal Surgery, The Affiliated Hospital of North Sichuan Medical College, Nanchong, Sichuan, People's Republic of China; ^2^ The Department of Pediatric Surgery, The Affiliated Hospital of North Sichuan Medical College, Nanchong, Sichuan, People's Republic of China; ^3^ The Department of HPB Surgery, Nanchong Central Hospital, The Second Clinical Medical College of North Sichuan Medical College, Nanchong, Sichuan, People's Republic of China; ^4^ The Department of General Surgery, Chengdu First People's Hospital, Chengdu, Sichuan, People's Republic of China

**Keywords:** long non-coding RNA, gastric cancer, SLC7A11-AS1, SLC7A11, proliferation

## Abstract

Many lncRNA and mRNA sense-antisense transcripts have been systematically identified in malignant cells. However, the molecular mechanisms of most lncRNA-mRNA pairs in gastric cancer remain largely unknown. We found the gastric cancer-associated lncRNA SLC7A11-AS1 and coding transcript mRNA SLC7A11 in human gastric cancer specimens by microarray. SLC7A11-AS1, antisense to SLC7A11, is significantly down-regulated in gastric cancer and could promote tumor growth *in vitro* and *in vivo*. The effects of SLC7A11-AS1 depend on the regulation of SLC7A11 via the ASK1-p38^MAPK^/JNK signaling pathway. These findings suggest that decreased expression of SLC7A11-AS1 contributes to the progression of gastric cancer and may be a novel diagnostic biomarker and effective therapeutic target in gastric cancer patients.

## INTRODUCTION

Gastric cancer (GC) is the second most lethal cancer globally. Although its incidence rate and mortality rate are decreasing, more than 24,590 people are diagnosed with GC each year, and 10,720 of them die from this type of cancer in USA [1]. Despite improvements in diagnosis and treatment strategies, the number of cases of survival is still low because the diagnosis tends to occur at an advanced stage [1, 2]. Therefore, the molecular characterization of gastric carcinogenesis and the identification of novel biomarkers for GC are urgently needed.

Long non-coding RNA (LncRNA) is a novel class of noncoding RNA that have lengths greater than 200 nucleotides (nt), lack significant open reading frames and can be placed into five broad categories [3]. Extensive evidence has revealed lncRNAs play a vital regulatory role in cancer biology through a variety of mechanisms affecting transcription levels and post-transcription levels [3–5]. Recently, remarkable progress concerning lncRNA effects on GC has been reported for both in cell behavior and clinicopathological factors [6]. For instance, CCAT1 [7], MALAT1 [8], and HOTAIR [9] are associated with clinicopathological factors, while GHET1 [10], TINCR [11] and PVT1 [12] all indicate that lncRNA is highly involved in the carcinogenesis of GC. However, the gastric cancer-associated aberrant signaling pathways remain unclear.

In a primary screening of the aberrantly expressed lncRNAs and mRNAs in GC, this study found that the lncRNA SLC7A11-AS1 was expressed at a lower level than its neighboring mRNA SLC7A11, which was highly expressed in the GC tissues examined. SLC7A11(xCT) is a regulatory light chain component of the cysteine/glutamate transporter whose expression on the cell surface is essential for the cellular uptake of cysteine and plays a role in intracellular glutathione (GSH) synthesis [13]; thus, SLC7A11 is an important determinant of the intracellular redox balance [14]. Cells deficient in SLC7A11 or depleted of GSH have been found to exhibit p38^MAPK^/JNK activation even at low levels of oxidative stress [15–17], indicating that SLC7A11 mediates cystine transport for GSH synthesis, plays a critical role in preventing of ROS-p38^MAPK^/JNK signaling and is implicated in the proliferation of several types of cancer cells.

SLC7A11-AS1 is located at the locus of chromosome 4q28.31, which has a SLC7A11 cis-natural antisense transcript (cis-NAT), but its clinical value and biological function in GC remain enigmatic. To study the possible role of SLC7A11-AS1 in GC, we carried out this study to compare SLC7A11-AS1 expression in GC tissues and their ANTs and PBMCs. We found that the expression of SLC7A11-AS1 was significantly decreased in the majority of gastric carcinoma and PBMCs and that reduced SLC7A11-AS1 expression was directly correlated with proliferation ability. In this study, we further analyzed the expression of SLC7A11-AS1 and its association with neighboring sense SLC7A11. We found that the expression of SLC7A11 was significantly increased in gastric carcinoma. Increased SLC7A11 in GC tissues prompted us to examine the biological role of SLC7A11-AS1 in GC via neighboring sense SLC7A11. Surprisingly, SLC7A11 was also increased in SLC7A11-AS1^low^ GC tissues compared with SLC7A11-AS1^high^ GC tissues. ASK1, cyclin D1 and Gclm expression levels also had similar trends. Upon further study, when SLC7A11-AS1 was knocked down in the SLC7A11-AS1^high^ GC cell lines, SLC7A11 was overexpressed. Moreover, SLC7A11-AS1 knock down was able to increase proliferation and promote cell cycle progression in tumor cells, as well as up-regulate Gclm, ASK1, c-Jun, and cyclin D1 expression and down-regulate p38 expression. Together, these results identify the critical role that SLC7A11-AS1 plays in the pathogenesis of GC via ASK1-p38^MAPK^/JNK signaling. Our findings provide supporting evidence for the regulatory roles played by lncRNAs in the progression of aggressive GC.

## RESULTS

### Identification of candidate gastric cancer-associated lncRNAs and coding transcript mRNAs

LncRNAs exhibit distinct expression patterns in tissues; however, we have limited knowledge of their precise molecular roles during mammalian development. To identify gastric cancer-relevant lncRNAs, the expression profiles of lncRNAs (Supplementary Figure 1A) and coding transcript mRNAs (Supplementary Figure 1B) were detected by microarray. A total of 4643 lncRNAs with a significant differential expression change of more than two-fold were identified: 2617 up- and 2026 down-regulated. A total of 3,944 coding transcript mRNAs that were differentially expressed by more than two-fold were identified: 1,948 mRNAs were up-regulated, and 1,996 mRNAs were down-regulated. We further analyzed the combined of the data concerning lncRNAs and mRNAs, based on lncRNA neighboring differential coding transcripts of mRNA expression profiles. We focused on the antisense lncRNA SLC7A11-AS1 (Supplementary Figure 2) and the neighboring sense coding transcript mRNA SLC7A11 (Supplementary Table 2) because the levels of SLC7A11 (located approximately 300 kb downstream of the lncRNA locus) were significantly changed.

### SLC7A11-AS1 is down-regulated in human GC tissues and PBMCs and is correlated with clinical-pathological factors in GC patients

**Figure 1:**
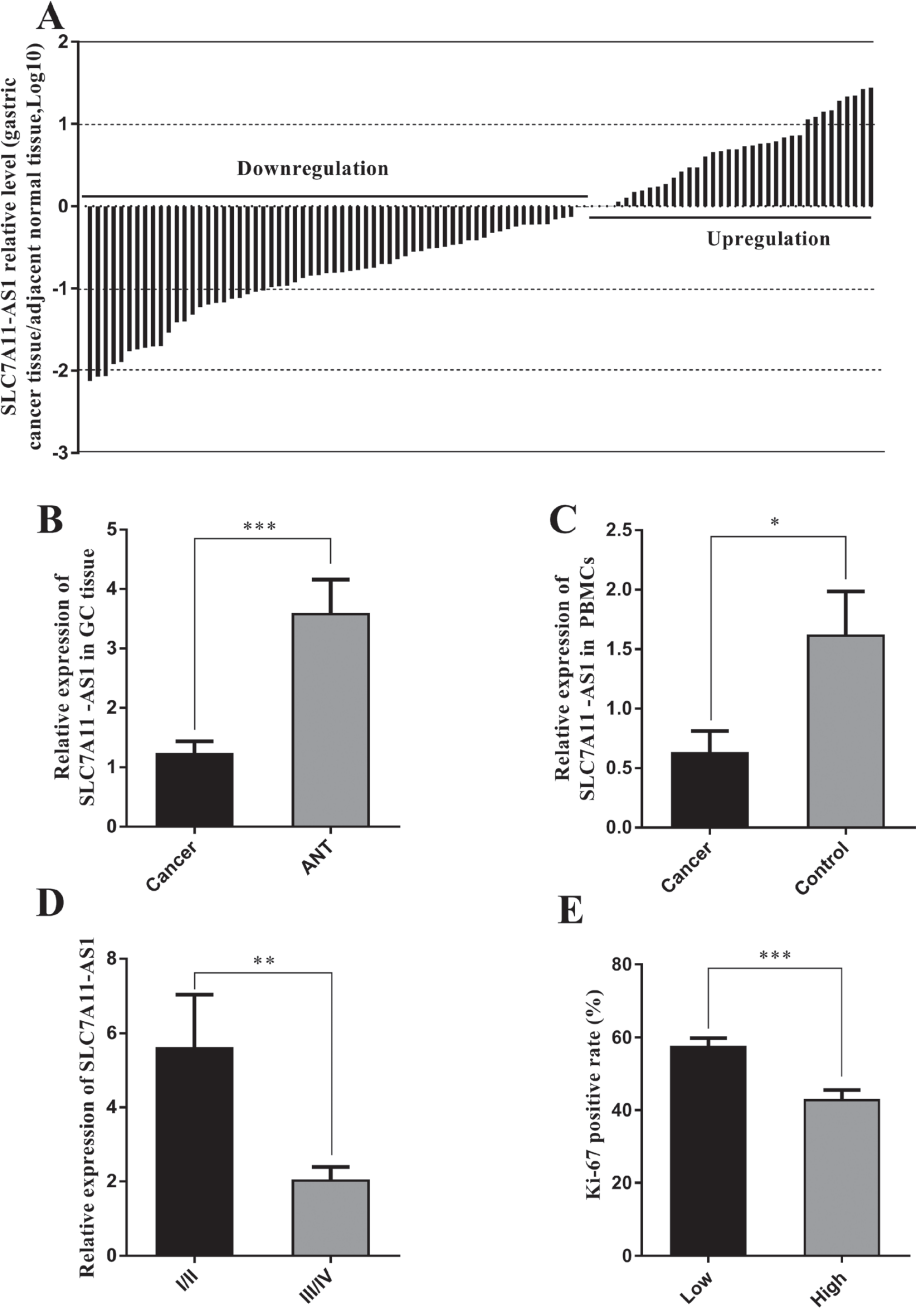
SLC7A11-AS1 expression levels were assessed in human gastric cancer tissues and PBMCs (**A**) SLC7A11-AS1 expression was assessed in human GC tissues and paired adjacent noncancerous tissues (ANTs). Bars represent the ratio between expression in GC tissues and ANT (C/N, log scale) from 100 patients was presented. GC tissues express significantly lower levels of SLC7A11-AS1 compared with paired ANTs in the majority of patients (64%). (**B**) The relative expression levels of SLC7A11-AS1 in GC tissues was significantly lower than those in ANTs (*P* < 0.001, *N* = 100). (**C**) The relative expression levels of SLC7A11-AS1 in 33 GC patients’ PBMCs was significantly lower than those in 20 healthy controls (*P* = 0.011). (**D**) SLC7A11-AS1 expression level was down-regulated in the stage III/IV GC tissues compared with the stage I/II GC tissues (*P* = 0.003). (**E**) Ki-67 positive rate was higher in the SLC7A11-AS1 low expression group (*N* = 48) than that in the SLC7A11-AS1 high expression group (*N* = 22) (*P* < 0.001). Expression levels were normalized to β-actin levels. Results are shown as mean ± SEM. (^*^P < 0.05, ^**^P < 0.01, ^***^P < 0.001, two-tailed Student's *t*-test).

**Figure 2:**
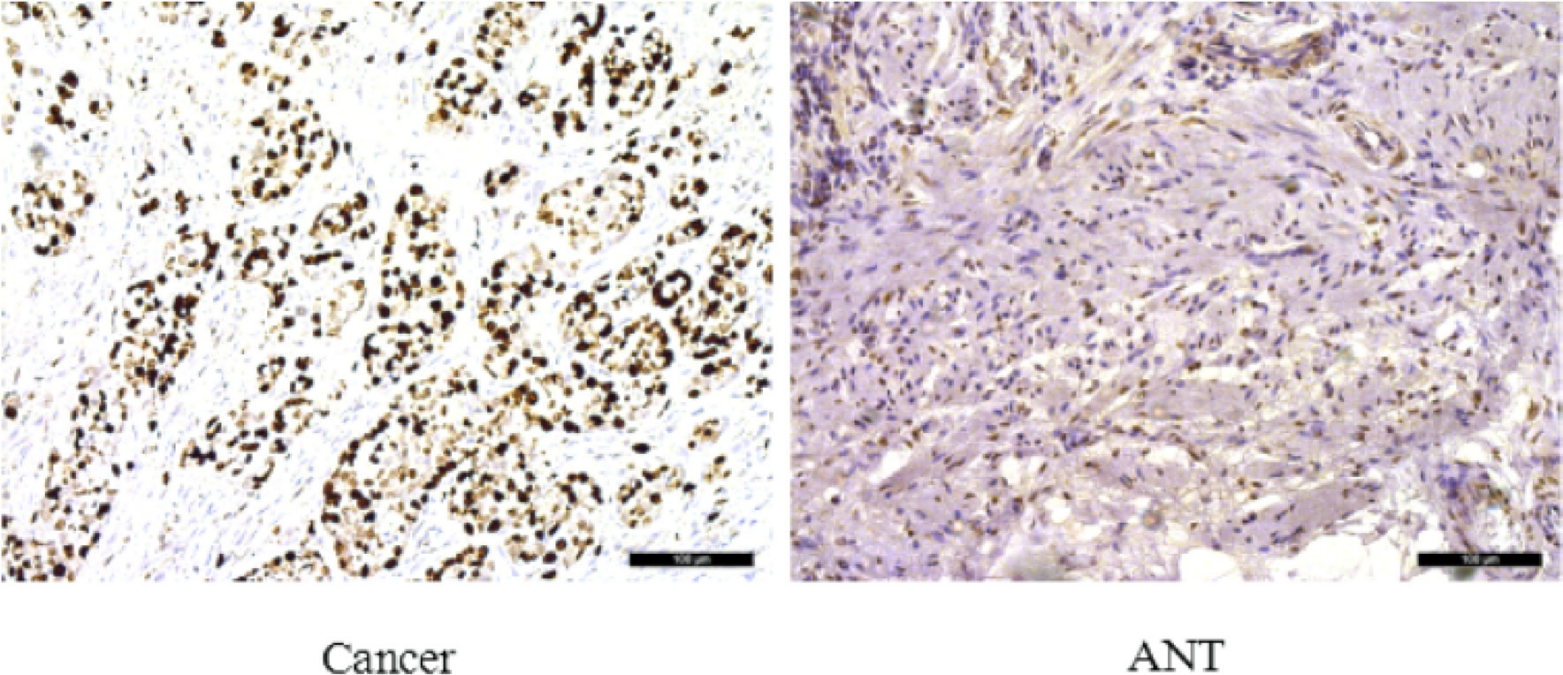
Immunohistochemical staining for Ki-67 in pairs of representative gastric cancer tissues with adjacent non-tumorous tissues (ANTs) Ki-67 labeling tumor cells were clearly identified by their brown nuclear staining. Ki-67 was mainly found in the nuclei of the carcinoma cells. High expression of Ki-67 in GC tissues compare with ANTs (200×). Scale bars, 100 mm.

To assess the role of SLC7A11-AS1 in GC progression, GC tissues and PBMCs were assayed. Decreased expression of SLC7A11-AS1 was detected in 64% of cancerous tissues compared with their noncancerous counterparts (Figure 1A), indicating that SLC7A11-AS1 expression was frequently decreased in cancerous tissues (Figure 1B). In addition, SLC7A11-AS1 levels were down-regulated in GC patients’ PBMCs (Figure 1C). When relationships between SLC7A11-AS1 expression and the clinicopathological features were investigated, the expression level of SLC7A11-AS1 was significantly associated with distant metastasis, macroscopic type and CEA. However, unfortunately, SLC7A11-AS1 expression levels were not associated with patient depth of invasion or lymph node metastasis, tumor sizes, cell differentiation, venous/lymphatic invasion, ascitic fluid, nervous invasion, or liver metastasis (Table 1). It is noteworthy that the expression levels of SLC7A11-AS1 were significantly associated with the GC tumor-node-metastasis (TNM) stage and Ki-67 positive rate, and significantly decreased expression of SLC7A11-AS1 was observed in the stage III/IV GC tissues compared with the stage I/II GC tissues (Figure 1D). In cancerous tissues, the Ki-67 positive rate in the SLC7A11-AS1^low^ expression group was higher than that in the SLC7A11-AS1^high^ group (Figure 1E). Immunohistochemical staining also shows that high expression of Ki-67 in cancerous tissues correlates with the ANTs (Figure 2). Together, these data suggest an important role for SLC7A11-AS1 in GC.

**Table 1:** Association between SLC7A11-AS1 expression and clinicopathological factors of human gastric cancer

Characteristics	NO. of each group	SLC7A11-AS1 expression		*P* value
		Low	High	
All case	100	64	36	
Age, (years, Mean ± SD)	100	61.67 ± 10.28	63.28 ± 8.9	0.434
Gender				0.656
Male	81	51	30	
Female	19	13	6	
Smoking				0.334
Yes	28	20	8	
No	72	44	28	
Drinking alcohol				
Yes	26	14	12	0.210
No	74	50	24	
BMI ( kg/m^2^, mean ± SD)	100	21.88 ± 3.18	21.73 ± 2.81	0.809
WBC (10^9/L, Mean ± SD)	100	6.29 ± 2.10	6.44 ± 3.23	0.782
RBC (10^12/L, Mean ± SD)	100	6.58 ± 19.03	3.77 ± 0.80	0.380
LY (10^9/L, Mean ± SD)	100	1.44 ± 0.51	1.36 ± 0.56	0.468
Hemoglobin (g/L, Mean ± SD)	100	115.47 ± 29.61	110.28 ± 26.17	0.383
CEA (μg/L, Mean ± SD)	100	8.39 ± 12.27	2.86 ± 2.86	0.015
CA19-9 (U/L, Mean ± SD)	100	74.41 ± 252.63	17.97 ± 38.44	0.084
TP (g/L, Mean ± SD)	100	66.48 ± 11.71	64.99 ± 7.36	0.491
ALB (g/L, Mean ± SD)	100	39.83 ± 5.16	38.91 ± 5.47	0.404
Maximum tumor diameter (cm, Mean ± SD)	100	3.97 ± 1.71	4.60 ± 3.02	0.256
AST (U/L, Mean ± SD)	100	26.82 ± 10.68	27.95 ± 7.35	0.576
ALT (IU/L, Mean ± SD)	100	17.63 ± 11.46	18.58 ± 9.52	0.675
TCH (mmol/L, Mean ± SD)	100	4.27 ± 1.19	3.99 ± 1.17	0.257
TG (mmol/L, Mean ± SD)	100	1.17 ± 0.76	1.15 ± 0.70	0.902
Ki-67	59	55.35% ± 16.99%	44.38% ± 12.09	0.009
ASA Grade				0.593
I	1	1	0	
II	86	53	33	
III	14	10	3	
Macroscopic types				0.024
Mass	6	1	5	
Ulcerative	16	8	8	
Infiltrative ulcerative	73	52	21	
Diffuse infiltrative	5	3	2	
Histology				
Undifferentiated	10	8	2	0.627
Poor differentiated	54	35	19	
Middle differentiated	23	14	9	
Well differentiated	13	7	6	
Depth of invasion				0.698
pT1	7	4	3	
pT2	12	8	4	
pT3	3	1	2	
pT4	78	51	27	
Lymph node metastasis				0.118
pN0	32	19	13	
pN1	19	16	3	
pN2	24	12	12	
pN3	25	17	8	
Distant metastasis				0.007
pM0	89	53	36	
pM1	11	11	0	
Tumor TNM stage				0.024
I	8	4	4	
II	26	14	12	
III	55	35	20	
IV	11	11	0	
Venous/Lymphatic invasion				0.482
Positive	9	7	2	
Negative	91	57	34	
Nervous invasion				0.651
Positive	5	4	1	
Negative	95	60	35	
Peritoneal metastasis				1.000
Absent	99	63	36	
Present	1	1	0	
Liver metastasis				0.479
Absent	97	61	36	
Present	3	3	0	
Ascitic fluid				0.123
Negative	81	51	30	
Rare	15	12	3	
Medium	4	1	3	
Fatty nodules				0.927
Positive	8	5	3	
Negative	92	59	33	

Abbreviations: BMI, Body Mass Index; WBC, White Blood Cell; RBC, Red Blood Cell; LY, Lymphocyte Count; TP, Total Protein; ALB, Serum Albumin; AST, Aspartate Aminotransferase; ALT, Alamine Aminotransferase; TCH, Total Cholesterol; TG, Triglyceride; ASA, American Society of Anesthesiology.

### Diagnostic value of SLC7A11-AS1 in human GC tissues and PBMCs

**Figure 3:**
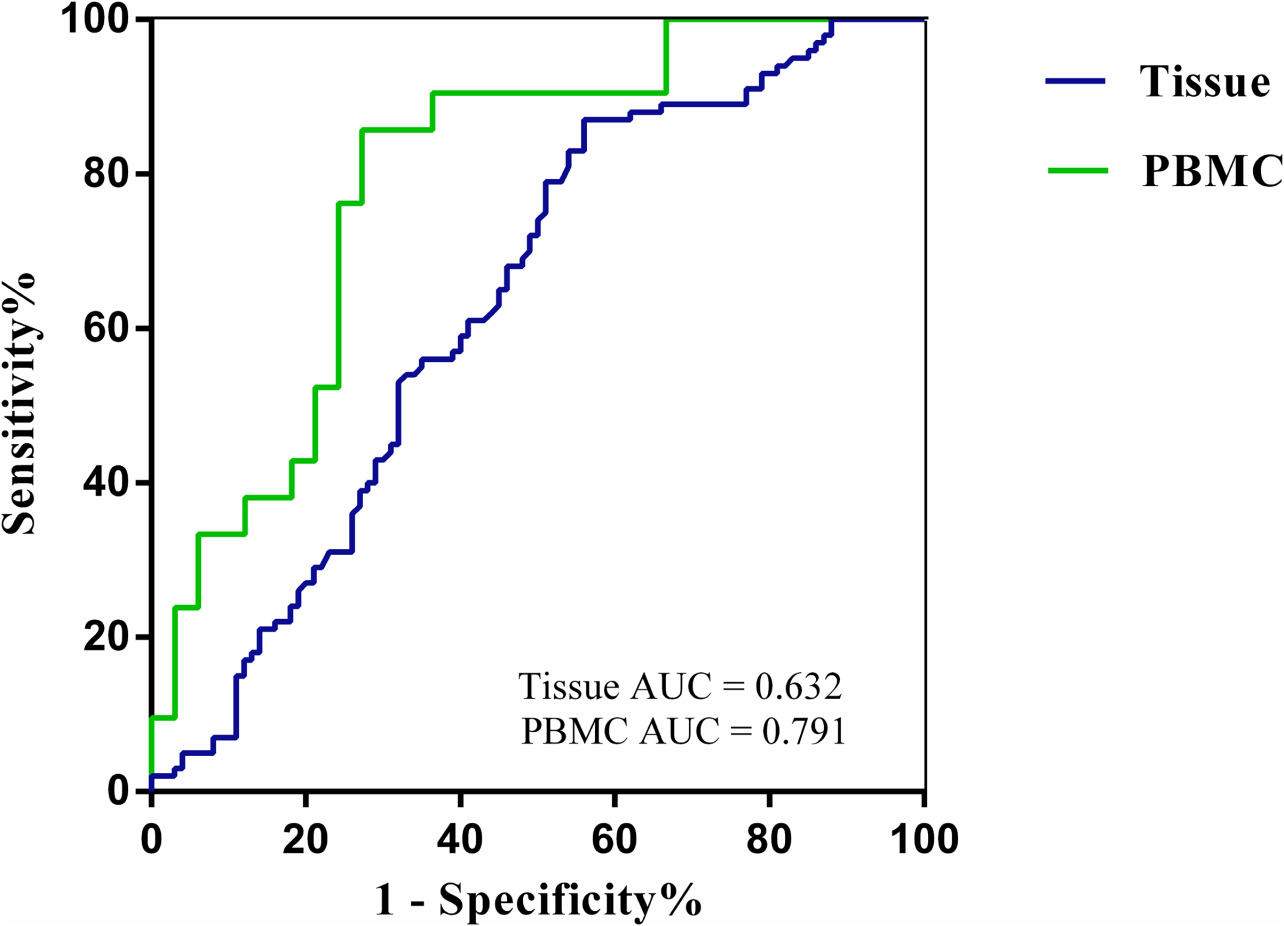
The ROC curves ROC curves analyses SLC7A11-AS1 for detecting gastric cancer patients in the tissues(AUC = 0.632, *N* = 100, *P* = 0.001,) and PBMCs (AUC = 0.791, *N* = 33, *P* < 0.001).

We observed whether SLC7A11-AS1 could be used as a marker of GC. A receiver operating characteristic (ROC) curve was generated by comparing SLC7A11-AS1 expression in GC tissues to expression in matched ANTs and in PBMCs in GC patients to expression in healthy controls (Figure 3). With a Youden index of 0.481, the area under the ROC curve (AUC) reached 0.791 (95% CI, 0.669 to 0.912); the sensitivity was 90.48%, and the specificity was 57.58% in PBMCs. Youden's index of 0.430 (95% CI, 0.554 to 0.710) was then used to identify an optimal cut-off point for the detection of GC, with a sensitivity of 87.00% and a specificity of 44.00%. The AUC curve was 0.632 for detecting SLC7A11-AS1 expression in tissue. These data suggest that SLC7A11-AS1 expression in the blood or tissues could serve as a molecular marker for GC.

### Reduced SLC7A11-AS1 levels are related to tumor proliferation in GC patients

To assess the role of SLC7A11-AS1 in GC progression, we analyzed some possible clinical factors related to tumor proliferation by univariate and multivariate logistic regression (Table 2). In the univariate analysis, tumor proliferation was related to drinking alcohol (OR, 5.200; 95%CI, 1.664 to 16.252) and SLC7A11-AS1 expression (OR, 4.250; 95%CI, 1.460 to 12.373). Furthermore, multivariate regression models demonstrated that the following factors increased the risk of tumor proliferation: drinking alcohol (OR, 4.485; 95%CI, 1.368 to 14.698) and SLC7A11-AS1 expression (OR, 3.634; 95%CI, 1.178 to 11.207). Taken together, these data suggest an important role for SLC7A11-AS1 in GC and indicate that abnormal SLC7A11-AS1 expression may be related to GC progression.

**Table 2:** Unitivariate and multivariate analyses for proliferation factors in gastric cancer patients

Factors	Univariate Analysis		Multivariate Analysis	
	OR (95% CI)	*P* value	OR (95% CI)	*P* value
Gender (male)	0.338 (0.085–1.346)	0.124		
Age, years	0.988 (0.938–1.041)	0.651		
BMI, kg/m^2^	1.000 (0.847–1.181)	0.998		
ASA Grade	1.097 (0.289–4.166)	0.892		
Tumor size, cm	1.167 (0.906–1.503)	0.232		
Smoking (Yes)	1.516 (0.523–4.395)	0.444		
Drinking alcohol (Yes)	5.200 (1.664–16.252)	0.005	4.485 (1.368–14.698)	0.013
Macroscopic types	1.441 (0.667–3.111)	0.353		
Depth of invasion	1.239 (0.735–2.089)	0.421		
Lymph node metastasis	0.991 (0.658–1.493)	0.967		
Distant metastasis	1.757 (0.361–9.760)	0.520		
Tumor TNM stage	1.783 (0.883–3.601)	0.107		
Ascitic fluid	1.761 (0.468–6.620)	0.403		
Histology (Well differentiated)	0.598 (0.309–1.158)	0.127		
Venous/Lymphatic invasion	3.649 (0.403–33.048)	0.250		
Nervous invasion	0.650 (0.086–4.906)	0.676		
Fatty nodules	0.417 (0.065–2.670)	0.356		
SLC7A11-AS1 (Low)	4.250 (1.460–12.373)	0.008	3.634 (1.178–11.207)	0.025

Abbreviations: OR, odds ratio; CI, confidence interval.

### SLC7A11 is up-regulated in human GC tissues and negatively correlated with SLC7A11-AS1 expression

**Figure 4:**
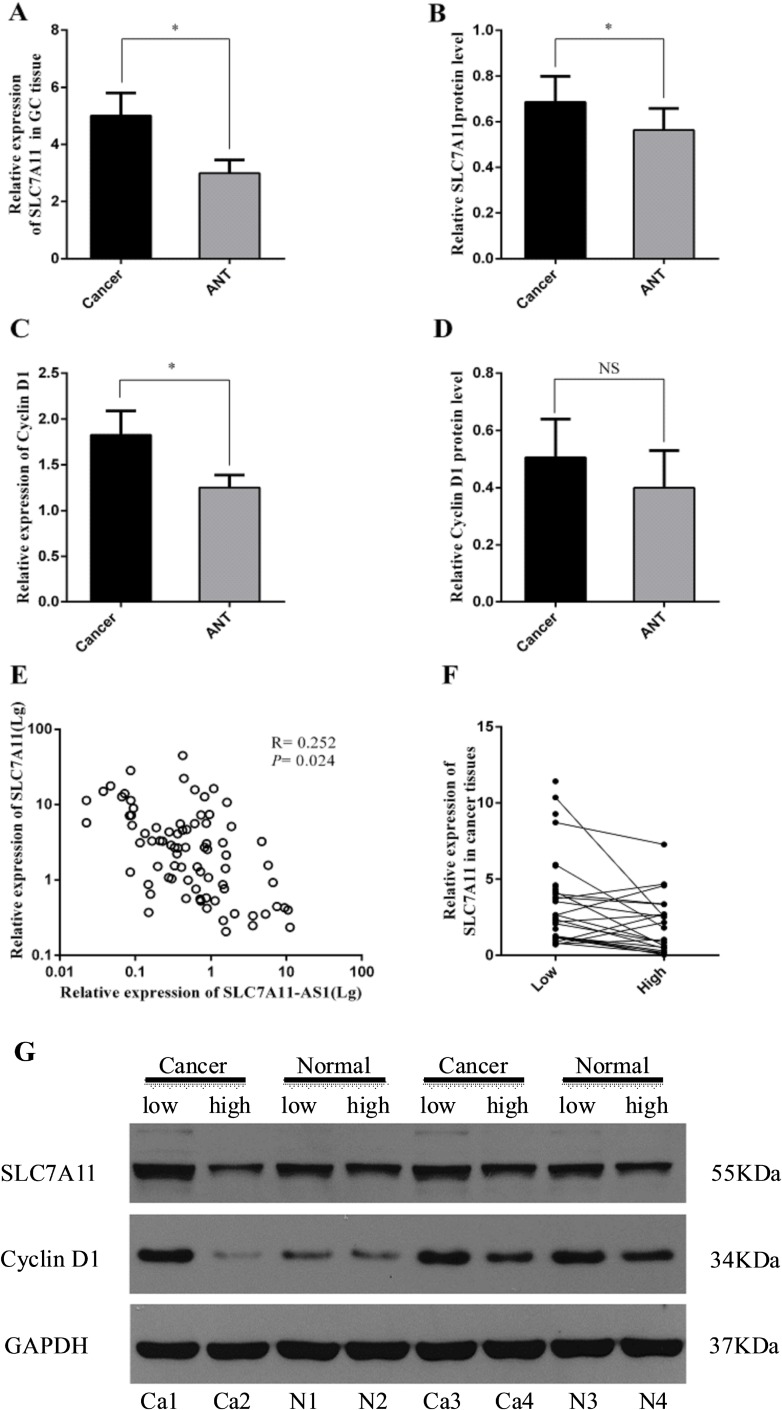
SLC7A11 and cyclin D1 expression level were assessed in human GC tissues (**A**) Levels of SLC7A11 in GC tissues was significantly higher than those in paired ANTs (*P* = 0.037, *N* = 80), expression levels were normalized to β-actin levels. (**B**). Western Blot analysis protein level of SLC7A11 between GC tissues and paired ANTs (*P* = 0.025, *N* = 75), expression levels were normalized to GAPDH levels. (**C**) Levels of cyclin D1 in GC tissues was significantly higher than those in paired ANTs (*P* = 0.040, *N* = 32), and expression levels were normalized to β-actin levels. (**D**) Western Blot analysis of protein level of cyclin D1 between GC tissues and paired ANTs (*P* = 0.385, *N* = 32), expression levels were normalized to GAPDH levels. (**E**) Bivariate correlation analysis of the relationship between SLC7A11-AS1 and SLC7A11 expression level, and the resulting Spearman correlation was calculated as 0.252 where *P* = 0.024 (*N* = 80). (**F**) SLC7A11 expression level was higher in the SLC7A11-AS1^low^ GC tissues than that SLC7A11-AS1^high^ GC tissues (*P* = 0.045), expression levels were normalized to β-actin levels. (**G**) SLC7A11 and cyclin D1 protein levels were increased in SLC7A11-AS1^low^ GC tissues compare with SLC7A11-AS1^high^ GC tissues. Results are shown as mean ± SEM. (^*^P < 0.05, NS: No significantly, two-tailed Student's *t*-test).

**Figure 5:**
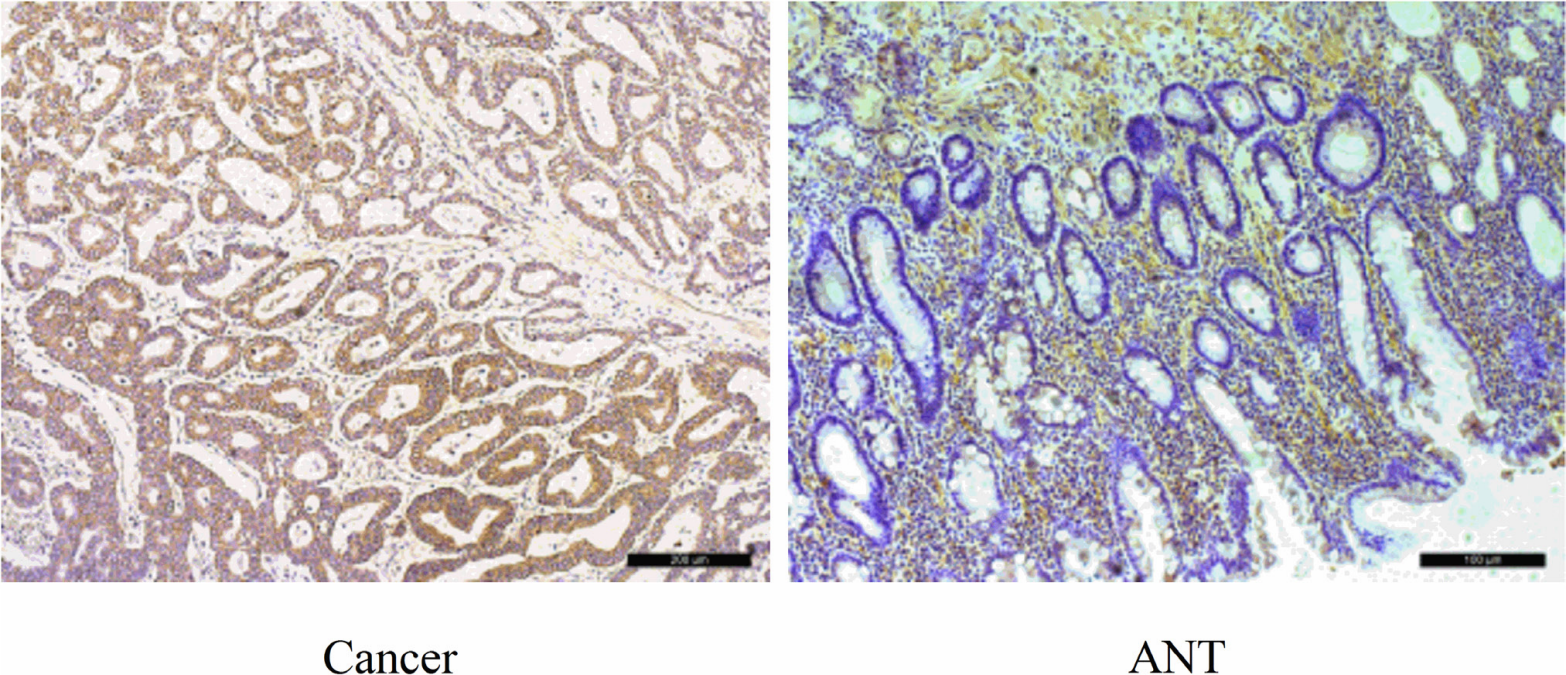
Immunohistochemical staining of SLC7A11 in pairs of representative gastric cancer tissues with adjacent non-tumorous tissues (ANT) Marked SLC7A11 expression was observed in GC. SLC7A11 was mainly found in the membrane of the carcinoma cells. High expression of SLC7A11 in GC tissues compare with ANTs (200×). Scale bars, 100 mm.

Because a considerable number of lncRNAs can function as a cis- or trans- regulator for their neighboring genes, the level of SLC7A11 in GC samples was also detected. As shown in Figure 4A and 4B, the mRNA and protein levels of SLC7A11 were up-regulated significantly in cancer tissue. Immunohistochemical staining also showed high expression levels of SLC7A11 in GC tissues compared with those in the ANTs (Figure 5). The correlation analysis indicates that a negative correlation existed between the expression levels of SLC7A11-AS1 and SLC7A11 in GC samples (Figure 4E). As shown in Figure 4G and 4F, SLC7A11 mRNA and protein levels were up-regulated in the SLC7A11-AS1^low^ cancer tissues compared with the SLC7A11-AS1^high^ cancer tissue or paired ANTs. Moreover, we also detected cyclin D1 mRNA and protein levels in the same way. Interestingly, cyclin D1 expression was significantly increased in cancer tissue compared with the ANTs in the SLC7A11-AS1^low^ cancer tissue groups (Figure 4C), and the protein level of cyclin D1 was up-regulated in the cancer tissue; unfortunately, there were no statistically significant differences (Figure 4D). However, it is noteworthy that cyclin D1 protein levels in the SLC7A11-AS1^low^ cancer tissues were higher than those in the SLC7A11-AS1^high^ cancer tissues. The correlation analysis also indicates that a negative correlation existed between the expression levels of SLC7A11-AS1 and cyclin D1 in GC samples (Supplementary Figure 3). According to these data, we speculated that the expression of SLC7A11-AS1 was also correlated with cyclin D1 in the GC samples.

### The biological effect of SLC7A11-AS1 relies on the regulation of SLC7A11

**Figure 6:**
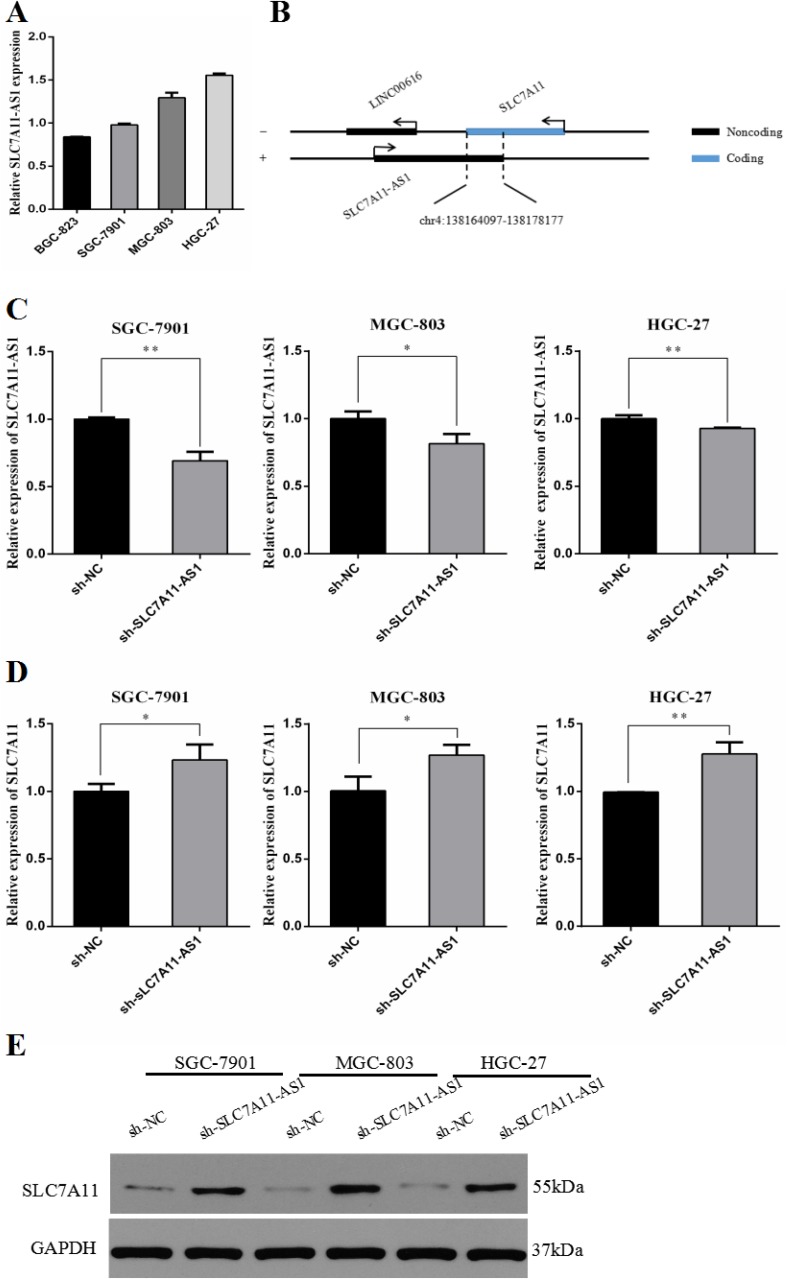
The expression of SLC7A11-AS1 and SLC7A11 were changed after transfection of SLC7A11-AS1 specific shRNA or negative control shRNA (**A**) The relative expression of SLC7A11-AS1 was measured in various cell lines were detected by qPCR, including 4 GC cell lines (SGC-7901, BGC-823, MGC-803 and HGC-27). (**B**) Schematic diagram was for exhibiting the location between SLC7A11-AS1 and SLC7A11. (**C**) SLC7A11-AS1 knocked down in SGC-7901 (*P*=0.001), MGC-803 (*P* = 0.023) and HGC-27 (*P* = 0.008) cells infected adenovirus shRNA by sh-NC and sh-SLC7A11-AS1 vector. (**D**) The SLC7A11 expression level was up-regulated in SGC-7901 (*P* = 0.034), MGC-803 (*P* = 0.025) and HGC-27 (*P* = 0.005) cells after knocked down SLC7A11-AS1. Expression levels were normalized to GAPDH levels. (**E**) The SLC7A11 protein level was up-regulated in SGC-7901, MGC-803 and HGC-27 cells after knocked down SLC7A11-AS1. Expression levels were normalized to GAPDH levels. Results are shown as mean ± SEM. (^*^P < 0.05, ^**^P < 0.01, two-tailed Student's *t*-test)

To determine whether the gene-proximal lncRNA SLC7A11-AS1 is typically correlated with the expression of the nearest mRNA SLC7A11, we first detected the expression of SLC7A11-AS1 in different GC cell lines (Supplementary Table 3). As shown in Figure 6A, very low levels of SLC7A11-AS1 were found in BGC-823. Oppositely, SGC-7901, MGC-803 and HGC-27 cells expressed significantly higher levels of SLC7A11-AS1. According to the relative expression of SLC7A11-AS1 in the GC cell lines, we knocked down SLC7A11-AS1 in the SLC7A11-AS1^high^ cancer cell lines (Supplementary Table 4), including SGC-7901, MGC-803 and HGC-27 (Figure 6C). SLC7A11 mRNA and protein up-regulation were detected under SLC7A11-AS1-knockdown conditions in SLC7A11-AS1^high^ cell lines (Figure 6D, 6E). On the other hand, human SLC7A11-AS1 and SLC7A11 had a notable overlapping complementary sequence region at chromosome 4q28.31 (Figure 6B), where the SLC7A11-AS1 gene contains five exons (3–7/7), and the SLC7A11 gene contains one exon (12/12). Together, these data suggest that the SLC7A11 gene may be a target of SLC7A11-AS1 that mediates expression.

### SLC7A11-AS1 ablation promotes GC development through increasing cell proliferation and cell cycle progression

**Figure 7:**
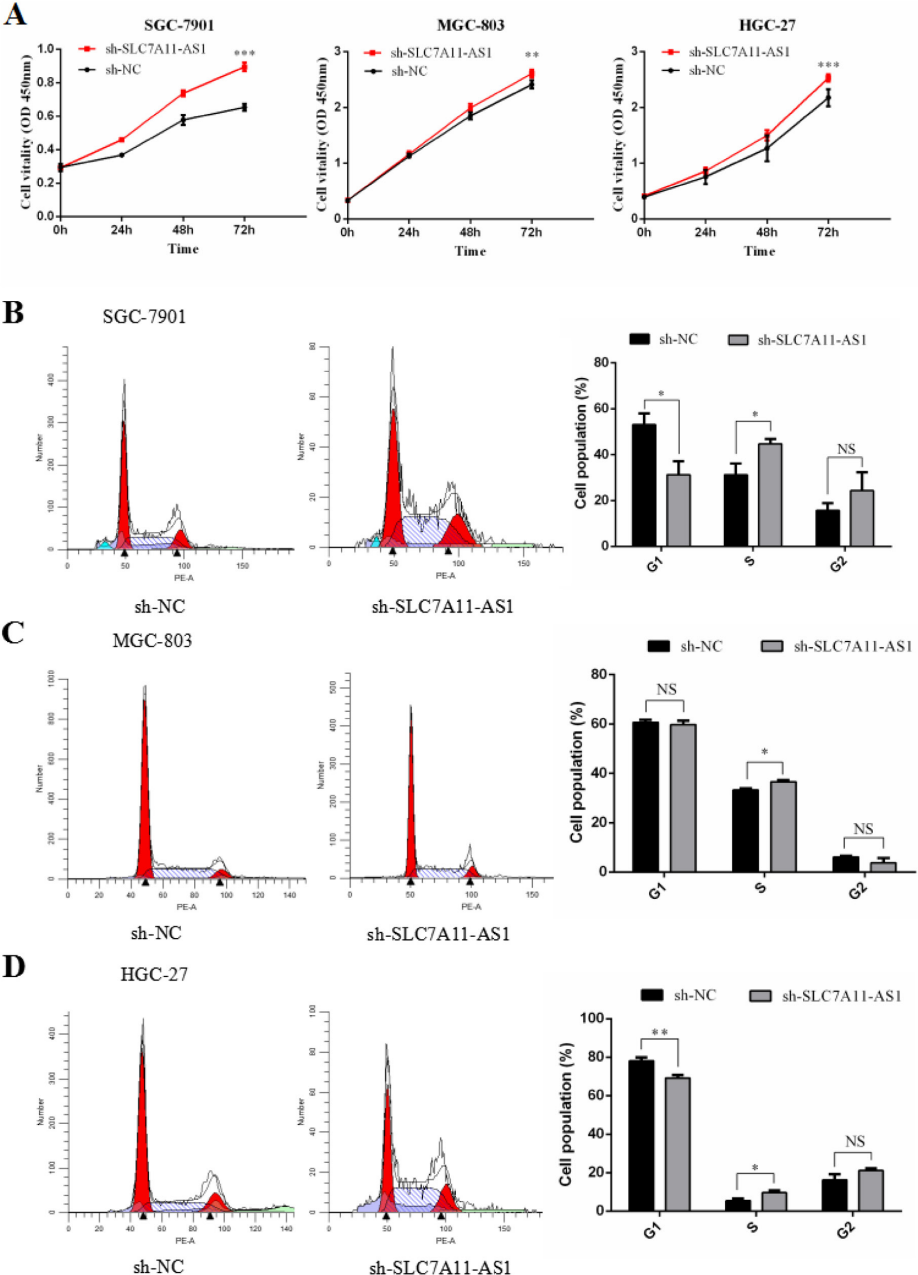
SLC7A11-AS1 low expression promotes gastric cancer cells proliferation and causes G1/S progress (**A**) The function of SLC7A11-AS1 on cell proliferation was determined by CCK8 assay. Cell proliferation was increased after knocked down SLC7A11-AS1 in SGC-7901 (*P* < 0.001), MGC-803(*P* = 0.046) and HGC-27(*P* < 0.001) cells. (**B**–**D**) The function of SLC7A11-AS1 on cell cycle was measured by flowcytometry after PI staining in SGC-7901, MGC-803 and HGC-27 cells. Error bars suggest mean ± SEM. (^*^P < 0.05, ^**^P < 0.01, ^***^P < 0.001, NS: No significantly).

To perform loss-of-function experiments, we generated adenoviral-based shRNAs targeting SLC7A11-AS1 which was previously identified in SLC7A11-AS1^high^ GC cancer cell lines. These shRNAs successfully targeted SLC7A11-AS1 and reduced its expression compared to that in the negative controls. Cell proliferation was significantly up-regulated (Supplementary Table 5) when SLC7A11-AS1 was functionally knocked down in SGC-7901, MGC-803 and HGC-27 cells (Figure 7A). Furthermore, when SLC7A11-AS1 was down-regulated, significant G1/S progression was observed (Supplementary Table 6) in SGC-7901, MGC-803 and HGC-27 cells (Figure 7B–7D). These data indicate that SLC7A11-AS1 functions as a GC suppressor by suppressing proliferation and arresting the cell cycle in GC cells.

### SLC7A11-AS1 functions as a GC suppressor through modulating the expression of SLC7A11 and activating ASK1-p38^MAPK^/JNK signaling

**Figure 8:**
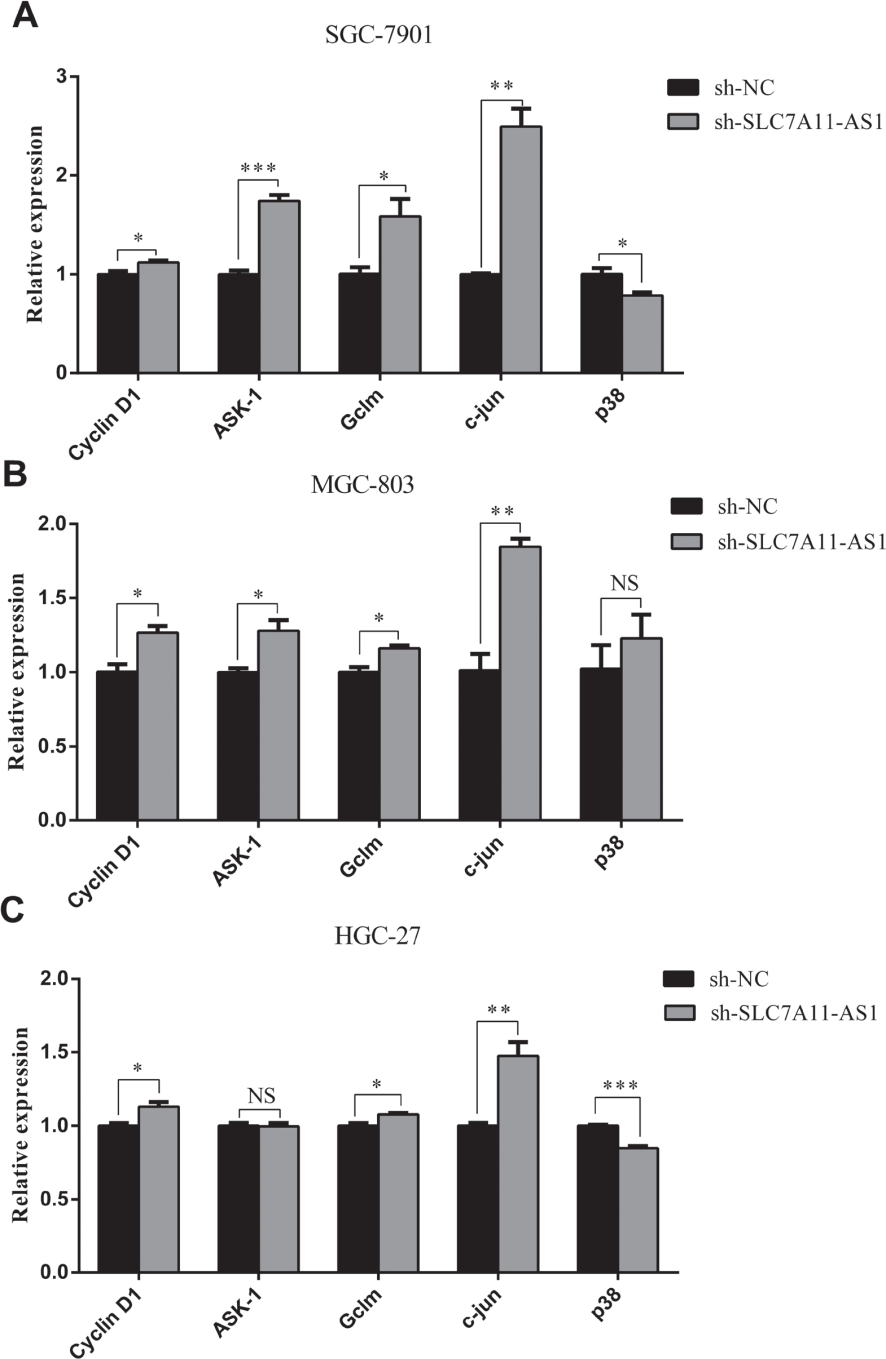
The expression of cyclin D1, ASK1, Gclm, p38 and c-Jun were changed after knocking down SLC7A11-AS1 with specific shRNA or negative control shRNA (**A**) The cyclin D1 (*P* = 0.035), ASK1 (*P* < 0.001), Gclm (*P* = 0.037), p38 (*P* = 0.032) and c-Jun (*P* = 0.001) expressed level in SGC-7901 cells when SLC7A11-AS1 was knocked down. (**B**) The cyclin D1 (*P*=0.018), ASK1 (*P* = 0.021), Gclm (*P* = 0.015), p38 (*P* = 0.415) and c-Jun (*P*=0.003) expressed level in MGC-803 cells when SLC7A11-AS1 was knocked down. (**C**) The cyclin D1 (*P* = 0.025), ASK1 (*P* = 0.826), Gclm (*P* = 0.008), p38 (*P* < 0.001) and c-Jun (*P* = 0.008) expressed level in HGC-27 cells when SLC7A11-AS1 was knocked down. Expression levels were normalized to GAPDH levels. Results are shown as mean ± SEM. (^*^P < 0.05, ^**^P < 0.01, ^***^P < 0.001, NS: No significantly, two-tailed Student's *t*-test).

**Figure 9:**
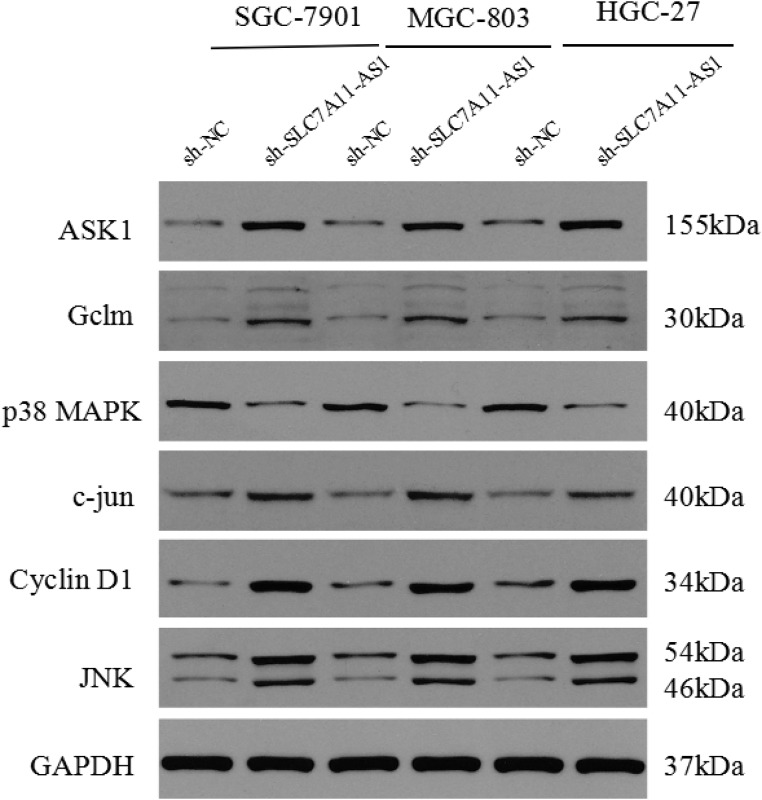
The protein of cyclin D1, ASK1, Gclm, p38, c-Jun and JNK were changed after knocking down SLC7A11-AS1 with specific shRNA or negative control shRNA The ASK1, Gclm,, c-Jun, cyclin D1 and JNK protein levels were up-regulated and p38 protein level was down-regulated in SGC-7901, MGC-803 and HGC-27 cells after knocking down SLC7A11-AS1, expression levels were normalized to GAPDH levels.

**Figure 10:**
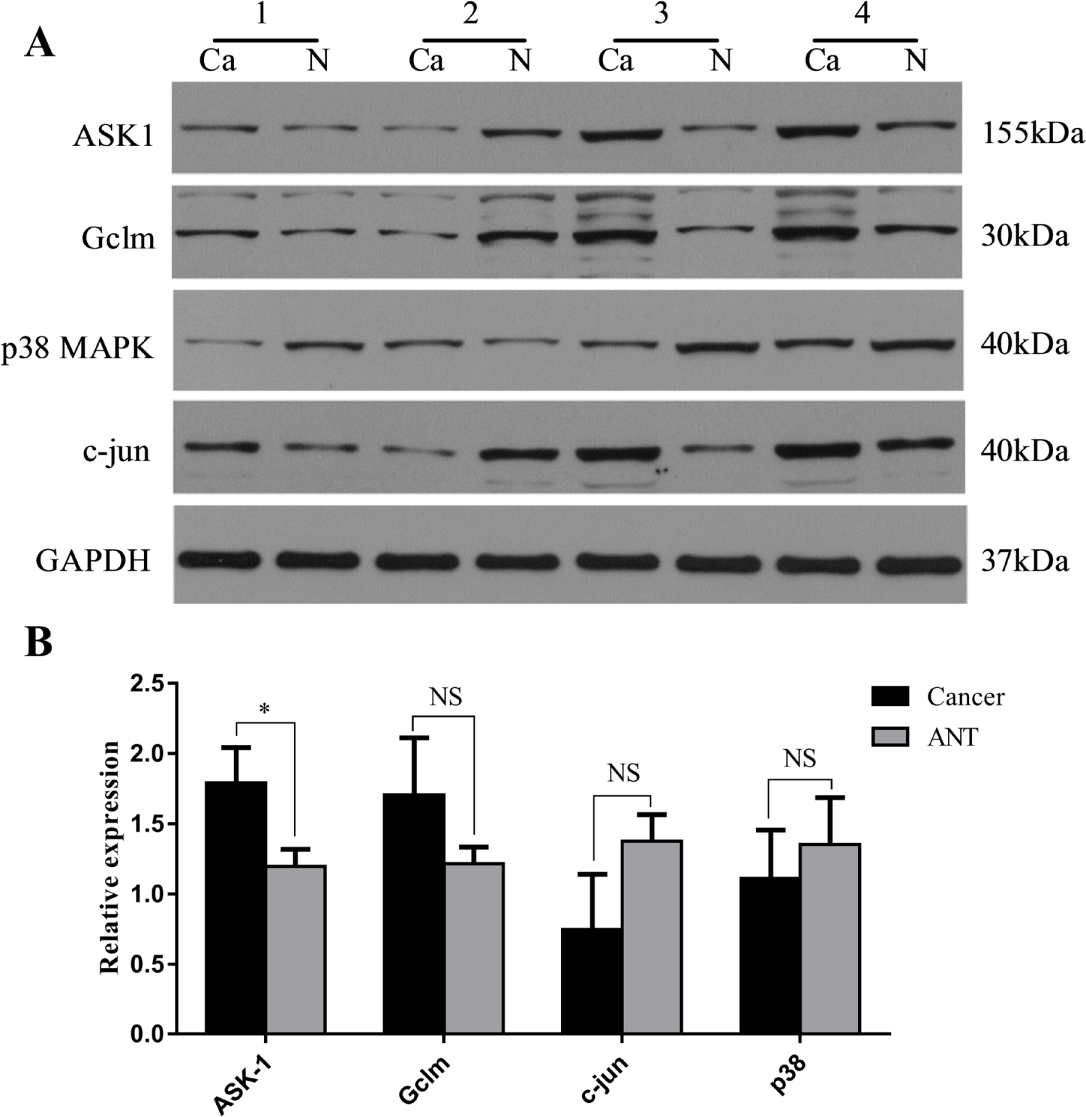
Expression changes of ASK1, Gclm, c-Jun and p38 were assessed in human SLC7A11-AS1^low^ gastric cancer tissues (**A**) The protein level of ASK1, Gclm and c-Jun were increased and p38MAPK was decreased in 30 paired SLC7A11-AS1^low^ GC tissues compared with ANTs, expression levels were normalized to GAPDH levels. (**B**) The expression of ASK1 (*P* = 0.027) and Gclm (*P* = 0.217) mRNA level were up-regulated and p38 (*P* = 0.638) and c-Jun (*P* = 0.193) mRNA levels were down-regulated in 30 paired SLC7A11-AS1^low^ group GC tissues and respective ANTs. Expression levels were normalized to β-actin levels. Results are shown as mean ± SEM. (^*^P < 0.05, NS: No significantly, Ca: cancer tissue, N: respective ANTs, two-tailed Student's *t*-test)

**Figure 11:**
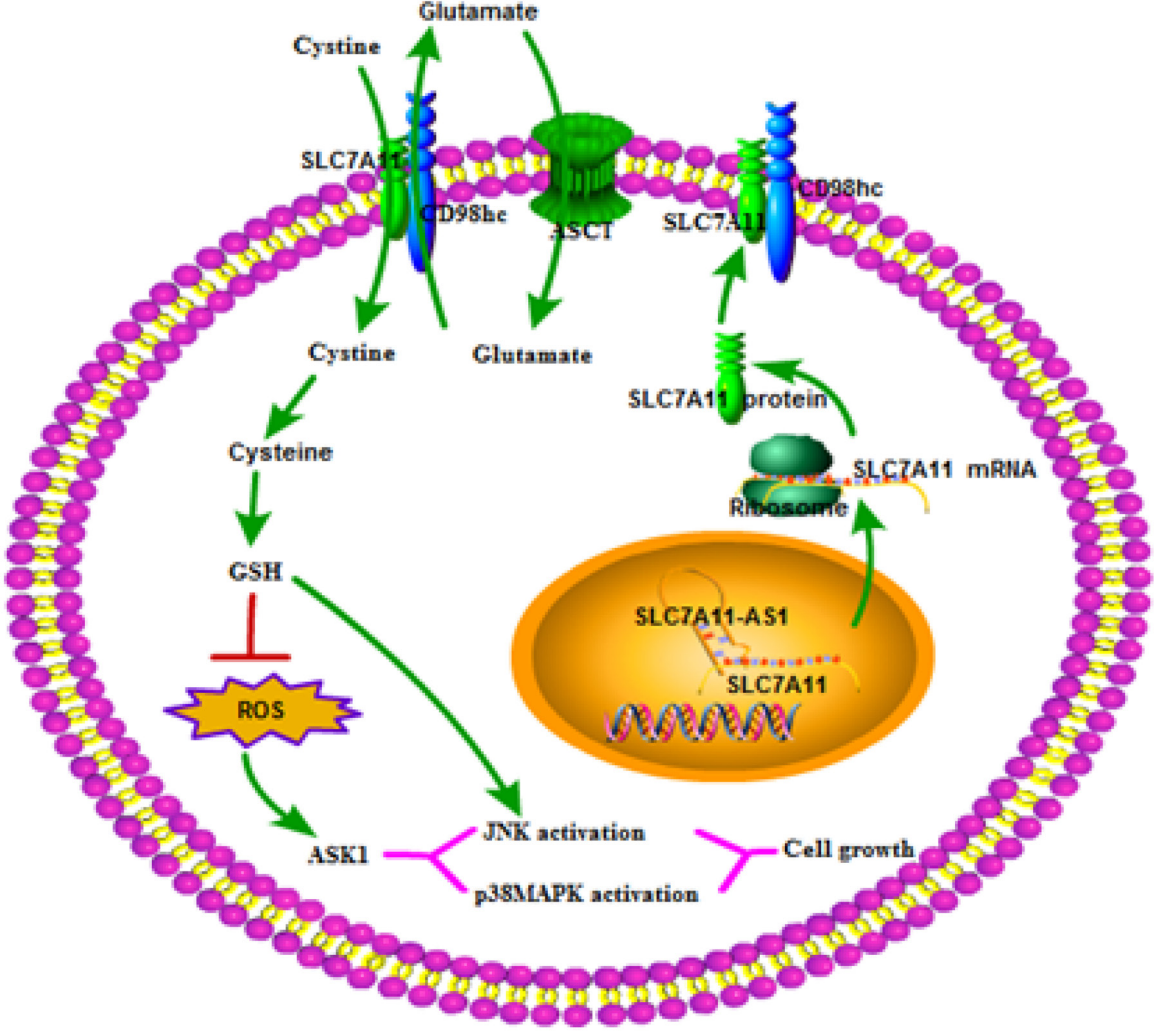
The role of the SLC7A11-AS1–SC7A11 model interacts in tumor development Decreased expression of SLC7A11-AS1 is increases GSH levels within cells by up-regulating SLC7A11 expression. The resultant increase in cellular GSH levels activates ASK1-p38^MAPK^/JNK signaling in cancer cells, thereby promoting tumor development. ASCT, system ASC glutamine transporter.

To further explore the mechanism though which SLC7A11-AS1 acts as a GC suppressor, we found clues to annotate the biological function of SLC7A11-AS1 through analyzing the data of the mRNA profile of SLC7A11 overexpression in SGC-7901, MGC-803 and HGC-27 cells when SLC7A11-AS1 was knocked down. The expression levels of Gclm, ASK1, c-Jun, and cyclin D1 were significantly up-regulated, and p38 expression was down-regulated when SLC7A11-AS1 was knocked down, but p38 and ASK1 were not significantly different in MGC-803 and HGC-27 cells, respectively (Figure 8A–8C). The protein levels of Gclm, ASK1, c-Jun, cyclin D1 and JNK were significantly up-regulated, and p38 was down-regulated when SLC7A11-AS1 was knocked down (Figure 9). In support of this finding, we also measured ASK1, Gclm, c-Jun, and p38 in 30 paired SLC7A11-AS1^low^ group GC tissues and their matched ANTs. The protein levels of ASK1, Gclm and c-Jun were up-regulated and p38 was down-regulated in GC tissues (Figure 10A). Nevertheless, we failed to detect a significant change in Gclm, c-Jun, and p38 expression levels; only ASK1 was up-regulated, suggesting that p38^MAPK^/JNK signaling was also regulated by another molecule *in vivo* (Figure 10B). These results indicate that SLC7A11-AS1 functions as a GC suppressor and that part of its mechanism of action may be related to ASK1-p38^MAPK^/JNK signaling (Figure 11).

## DISCUSSION

The relevance of noncoding RNAs, including microRNAs, lncRNAs and pseudogenes, in normal physiological processes and in tumorigenesis has been increasingly recognized [18]. LncRNAs exhibit distinct expression patterns in tissues; however, we have limited knowledge of their precise molecular roles during GC development. To discover new biomarkers and candidate compounds for GC diagnosis and treatment, we used a custom lncRNA microarray platform with probes for gastric cancer-related lncRNAs and protein-coding genes expressed in the human genome in GC. Based on the microarray results, we further analyzed the combined data from the lncRNAs and their neighboring differential mRNAs. Then, we selected SLC7A11-AS1 and its neighboring coding transcript SLC7A11 and investigated their expression levels in human GC tissues and their matched ANTs. SLC7A11-AS1 expression had a significantly low level in PBMCs and was down-regulated in GC and high stage cancer tissues. We also demonstrated that there were significantly more Ki-67 positive cells with low SLC7A11-AS1 expression than those with high SLC7A11-AS1 expression in patients. Therefore, SLC7A11-AS1 might play an important role in proliferation and tumor progression during GC development.

Furthermore, patients with lower SLC7A11-AS1 levels appeared to have a greater level of CEA, easier distant metastasis, and a higher tumor stage than the higher SLC7A11-AS1 group. We also demonstrated that significantly less SLC7A11-AS1 expression was detected in the peripheral blood of GC patients, compared with that observed in the healthy control group. ROC evaluation indicated that the blood SLC7A11-AS1 level would be a more sensitive marker than CEA and CA19-9 in GC diagnosis. At the same time, logistic regression confirmed that SLC7A11-AS1 may be a proliferation factor in GC. Similarly, recent studies have reported that aberrant expression levels of many other lncRNAs have been regarded as prognostic indicators in GC [19–21]. Our work on SLC7A11-AS1 suggests that it might function as a suppressor gene that provides a significant predictive value and may serve as a promising tumor marker for GC diagnosis.

LncRNAs are a group of versatile physiological mediators, and some of them have been shown to regulate the transcription of neighboring genes on the same chromosome, mainly through cis mechanisms [22, 23]. For instance, X-chromosome inactivation relies on the expression of a lncRNA Xist, which is thought to recruit protein complexes to establish repressive epigenetic marks that encompass the chromosome in a cis-specific manner [24]. In addition, the Xist and Tsix RNA pair serves as a paradigm for understanding sense-antisense relationships in eukaryotes [25]. Similarly, the depletion of lncRNA-a1 adjacent to ECM1 and lncRNA-a7 adjacent to Snai1 resulted in a concomitant decrease in the expression of neighboring ECM1 and Snai1 levels, respectively [26]. Inspired by these examples, we hypothesized that similar to the lncRNAs function of imprinting loci, SLC7A11-AS1 exerts a similar function on its neighboring gene SLC7A11. This relationship provided us with an opportunity to determine SLC7A11-AS1 function in cellular proliferation through the SLC7A11 expression changes. To confirm this hypothesis, we first detected SLC7A11 and cyclin D1 levels in cancer tissue. Amazingly, SLC7A11 and cyclin D1 were up-regulated in the cancer tissues at both the mRNA and protein levels, and the same trend was seen in the SLC7A11-AS1^low^ cancer tissues compared to the SLC7A11-AS1^high^ cancer tissues. Next, we depleted lncRNA SLC7A11-AS1 in GC cells, which led to an increase in SLC7A11 and cyclin D1 transcript and protein levels.

To further validate the function of lncRNA SLC7A11-AS1 in accordance with the above evidence, we used RNA interference to deplete SLC7A11-AS1 and assessed the effect on proliferation in the GC cell lines. Functional knockdown of lncRNA SLC7A11-AS1 also revealed its positive influence on cell proliferation in three GC cell lines. Moreover, depletion of SLC7A11-AS1 also increased SLC7A11-AS1-induced cyclin D1 and promoted cell cycle progression. But, SLC7A11-AS1 has different effects on the biological behavior of three GC cell lines, this is due to the heterogeneity of tumor cells. Altogether, these results demonstrate a role for SLC7A11-AS1 in cell proliferation and cell cycle progression and as a tumor suppressor gene. Despite these findings, our knowledge of SLC7A11-AS1 functions with SLC7A11 associated with cell proliferation is limited.

To identify whether SLC7A11 is involved in the regulation of proliferation by SLC7A11-AS1-induced signaling, we examined the effect of overexpression on the activity of signaling pathways associated with cell cycle progression and proliferation. SLC7A11 is a member of the system xc^-^ light chain family and whose overexpression directly affects AP-1 activation and cell cycle progression due to increased GSH levels caused by increased intracellular thiol reducing equivalents, which are necessary to allow cell cycle progression [27]. The mostly common forms of AP-1 consist of heterodimers of Jun and Fos family members [28], and among the Jun proteins, c-Jun is unique in its ability to positively regulate cell proliferation through the induction of cyclin D1 transcription that promotes cell entry into S-phase [29]. Herein, depletion of SLC7A11-AS1 had an effect on c-Jun and cyclin D1 overexpression that was related to tumor cell cycle progression, indicating that SLC7A11-AS1 regulated cell cycle and proliferation cause to SLC7A11 up-regulation by the c-Jun N-terminal kinase (JNK) and p38 pathways. Furthermore, apoptosis signal-regulating kinase 1 (ASK1) is a mitogen-activated protein kinase (MAPK) kinase kinase that activates the downstream MAPKs, JNK and p38 [30]. Thus, the expression of ASK1 and p38 might support cell proliferation and cell cycle progression when SLC7A11-AS1 is knocked down. We have shown that SLC7A11-AS1 ablation suppressed gastric tumor cell growth concomitant with the up-regulation of ASK1 and its downstream genes. However, the expression of ASK1 is not significantly changed in HGC-27 cells, and the p38^MAPK^ level was down-regulated in SGC-7901 and HGC-27 cells; moreover, the ASK1 mRNA level did not change in MGC-803 cells when SLC7A11-AS1 was knocked down. Notably, the dual roles of p38^MAPK^ in cell proliferation depend on the cell type and stimulus [31], and p38 has an inhibitory role in cell cycle progression that is correlated with inhibition of the serum-induced expression of cyclin D1, as well as a reduction in the expression and an activation of several cyclins and cyclin-dependent kinases [32–34]. These results may explain why the heterogeneous of tumor cells from different cell lines have the different main regulatory mechanisms due to different genetic backgrounds or these effects may mainly influence translation. Moreover, SLC7A11 carries out an essential step in the synthesis of GSH, and the modulatory subunit of glutamate cysteine ligase (Gclm), the rate-limiting enzyme of GSH synthesis, is up-regulated when SLC7A11-AS1 is knocked down. Therefore, it is possible that Gclm contributes to cell proliferation by regulating GSH levels. On the other hand, the protein levels of ASK1, Gclm, c-Jun, and p38 were up-regulated, but the expression levels of Gclm, c-Jun, and p38 did not change in the SLC7A11-AS1^low^ group GC tissues and their matched ANTs. These results suggest that gene expression and signaling pathways can be modulated by multiple ways *in vivo* and that these effects may mainly influence translation. Cumulatively, these findings indicate that SLC7A11-mediated suppression of ASK1-p38^MAPK^/JNK signaling that negatively regulates the cell cycle and proliferative capacities of tumor cells might play a role in SLC7A11-AS1-mediated tumor cells.

Widespread sense-antisense (SA) transcripts have been systematically identified in mammalian cells [35], and global transcriptome analyses show that up to 70% of transcripts have antisense partners and that perturbation of antisense RNA can alter the expression of the neighboring sense gene in complex loci into chains of linked transcriptional units [36]. SA pairing of transcripts is a common mode of gene regulation in the human genome; antisense genes tend to be inversely expressed with their sense partners and antisense regulation is as an important mechanism of gene regulation in the human genome [37]. We propose that lncRNA SLC7A11-AS1 expression is negatively regulated by its neighboring protein-coding SLC7A11 and that this effect is not specific to any one locus and may represent a general function of lncRNAs in mammalian cells.

SLC7A11-AS1 is mapped to chromosome 4q28.31, which is a SLC7A11 cis-NAT and it negatively regulates the expression of SLC7A11. Our results contrast the above mentioned studies that concluded that SLC7A11-AS1 and SLC7A11 SA pairs presented with tail-to-tail (3′ to 3′) overlap and acted via antisense regulation. The regulatory mechanisms by which SA acts are diverse; coupled SA expression was more prevalent in tail-to-tail NAT pairs, suggesting that such an orientation is not only the most abundant but also more likely to have a regulatory function [38]. Although the exact mechanism by which our lncRNA functions to enhance gene expression in antisense regulation remains unclear, it might be expected that there is an imbalance between the expression of sense and antisense genes under disease conditions, which would be modulated by antisense regulation of the pathogenesis of GC. These findings strongly support the hypothesis that antisense regulation is a common and important mechanism used in the human genome.

In conclusion, our present data provide evidence that the decreased expression of SLC7A11-AS1 and its association with SLC7A11 promote the growth of GC cells via ASK1-p38^MAPK^/JNK signaling and thereby promote the growth of cancer cells and the formation of lethal gastrointestinal tumors. On the other hand, given that SLC7A11-AS1 may have numerous functions, further investigation of other functions of this molecule in tumor growth and maintenance is needed.

## MATERIALS AND METHODS

### Patients and tumor tissues

Human GC tissues and the paired adjacent non-cancerous tissues (with a distance of 5 cm away from the tumor margin) of 100 patients were obtained at the time during surgery between February 2015 and June 2016 at the Affiliated Hospital of North Sichuan Medical College (Sichuan, China) and detailed clinical pathologic parameters. Following removal, the GC and non-cancerous tissues were immediately frozen in liquid nitrogen and stored at −80°C until used. PBMCs were isolated from 33 GC patients before surgery and 20 healthy volunteers. All patients and volunteers involved in the study gave written consent for the medical study, and all evaluations were performed voluntarily. The entire study's protocol was approved by the Ethics Committee of the Affiliated Hospital of North Sichuan Medical College, Nanchong, China.

### Microarray analysis

Three pairs of GC tissues and ANT were obtained from GC patients. RNA quantity and quality were measured by NanoDrop ND-1000 (Thermo Fisher, USA). RNA integrity was assessed by standard denaturing agarose gel electrophoresis. Human lncRNA microarrays were performed according to the Agilent One-Color Microarray-Based Gene Expression Analysis protocol (Agilent Technologies) with minor modifications; approximately 30,586 lncRNAs and 26,109 coding transcripts could be detected. Agilent Feature Extraction software (version 11.0.1.1) was used to analyze the acquired array images. Quantile normalization and subsequent data processing were performed by using the GeneSpring GX v12.1 software package (Agilent Technologies). Differentially expressed lncRNAs and mRNAs with statistical significance between the two groups were identified by using *P*-value/FDR filtering. Differentially expressed lncRNAs and mRNAs between the three sample types were identified through Fold Change filtering. Hierarchical clustering and combined analysis were performed using homemade scripts.

### Cell lines and cell culture

The human GC cell lines SGC-7901, BGC-823, MGC-803, and HGC-27 were purchased from Shanghai Cell Bank (China). All the cell lines have been tested and authenticated by Shanghai Cell Bank. The cells were cultured at 37°C in a humidified atmosphere of 5% CO_2_ in DMEM (Gibco-BRL, Gaithersburg, USA) supplemented with 10% fetal bovine serum (Gibco-BRL, Gaithersburg, USA) and antibiotics (100 IU/ml penicillin and 100 μg/ml streptomycin).

### Reverse transcription quantitative PCR

Total RNA was extracted from GC tissues and their ANTs using Trizol reagent (Invitrogen, Karlsruhe, Germany), and reverse transcription reactions were performed using the GoScript Reverse Transcription (RT) System (Promega, Madison, USA) according to the manufacturer's instructions. Real-time PCR was performed using a standard SYBR Green PCR kit (Roche, USA) and a Roche Light^®^ Cycler Instrument (Roche, USA) according to the respective manufacturer's instructions. β-actin or GAPDH was used to normalize lncRNAs. Each sample was analyzed in triplicate. Primers for SLC7A11-AS1, SLC7A11, ASK1, cyclin D1, Gclm, c-Jun, p38, β-actin and GAPDH were synthesized by Sangon Biotech (Shanghai, China), and their sequences are listed in Supplementary Table 1. The thermal cycling consisted of a denaturation step at 95°C for 10 min, followed by 40 cycles of denaturation at 95°C for 30 s, annealing at 63°C or 60°C for 30 s, and extension at 72°C for 60 s. At the same time, β-actin or GAPDH was amplified for normalization of the relative levels of lncRNA. Amplified DNA products were subjected to separation by 1.5% agarose gel electrophoresis and staining with ethidium bromide for visualization. The 2^-ΔΔCt^ method was used to quantify the relative levels of gene expression.

### Adenoviral infection

Cells were transiently infected with sh-SLC7A11-AS1 and non-specific sh-Negative Control (sh-NC) (8 μg/ml) transfection reagent (Hanbio, Shanghai, China) according to the manufacturer's instructions. The specific shRNA oligo for SLC7A11-AS1 was made by Hanbio (Shanghai, China). Two human SLC7A11-AS1-specific sequences (GenBank accession number NR_038380.1) were used; shRNA: 5′-AATTCGAACTGGTACCAACCTACTTACGAATTC AAGAGATTCGTAAGTAGGTTGGTACCAGTTCTTT TTTG-3′ or shRNA: 5′-AATTCGAGGTGTCTGTGAG GGTGTTTCTGTATTCAAGAGATACAGAAACACCC TCACAGACACCTTTTTTTG-3′. Cells were seeded at an 80% density in 6-well plates before infection, and the culture media was changed 6 h after infection. After infection for 48 h, the infected cells were harvested for the extraction of total RNA.

### Western blot

Total protein samples were extracted and the concentrations were determined using a BCA protein assay kit (Thermo Fisher Scientific, Waltham, MA, USA). Sample lysates (10 μg of protein) were separated by SDS-PAGE and transferred onto a PVDF membrane. The membrane was incubated with specific antibodies for SLC7A11 (1:1,000) or cyclin D1 (1:3,000; Abcam, Cambridge, MA, USA) at 4°C overnight followed by incubation with secondary antibodies. Protein levels were normalized to those of total GAPDH using a monoclonal anti-GAPDH antibody (Sigma-Aldrich Corporation, St. Louis, MO, USA). Autoradiograms were quantified by densitometry (Quantity One software; Bio-Rad).

### Immunohistochemistry

Paraffin-embedded, formalin-fixed tissues were immunostained for SLC7A11 and Ki-67 proteins using the avidin-biotin-peroxidase method. Briefly, paraffin sections were dewaxed in xylene and dehydrated using a graded series of alcohols. The sections were then treated with protein blockers and incubated overnight at 4°C with anti-SLC7A11 (1:200; Abcam, Cambridge, MA, USA) or anti-Ki-67 antibodies (1:100; Abcam, Cambridge, MA, USA). The signal was amplified and visualized with diaminobenzidine chromogen, followed by counterstaining with hematoxylin. Finally, the sections were dehydrated using a graded series of alcohols, cleared in xylene, and mounted. Tumor cells with cytomembrane and nuclei containing brown immunoreactive products were considered to be positive for SLC7A11 and Ki-67 expression, respectively.

### Cell proliferation assays

Cell proliferation was assessed using a Cell Counting Kit-8 assay (CCK-8, Beyotime Institute of Biotechnology, Shanghai, China) according to the manufacturer's instructions. For the CCK-8 assay, a total of approximately 1 × 10^4^ cells per well were seeded in a 96-well plate for 24 hours and then infected with the adenovirus shRNAs. After transfection for 48 h, the infected cell proliferation curves were plotted using the absorbance at each time point. All experiments were performed in triplicate.

### Flow cytometry assay

Cells were harvested after adenovirus infection for 48 h and then washed twice with cold PBS and fixed in 70% alcohol at −20°C overnight. After fixation, cells were stained with propidium iodide (PI) at a final concentration of 50 ng/mL in the dark at 37°C for 30 min to detect cell cycle. The stained cells were then analyzed by using flow cytometry (BD FACS Canto II, BD, USA). The flow cytometry assay was repeated three times.

### Statistical analysis

All statistical data were analyzed with Statistical Program for Social Sciences (SPSS) 13.0 software (SPSS, Chicago, IL) and GraphPad Prism 6.0 (GraphPad Software, La Jolla, CA). The differences between groups were analyzed using Student's *t*-test, Chi-Square test or the Fisher Exact test. Logistic regression analyses were done with 95% confidence interval and were performed by using the univariate and multivariate model to identify factors associated with tumor proliferation. A receiver operating characteristic (ROC) curve was established to evaluate the diagnostic value in cancer tissues and PBMCs. Spearman correlation analyses were performed to investigate correlations between gene expression levels. All the tests were two-tailed, and *P* < 0.05 was considered statistically significant.

## SUPPLEMENTARY MATERIALS FIGURES AND TABLES



## Abbreviations

GC: gastric cancer
LncRNA: long non-coding RNA
PBMCs: peripheral blood mononuclear cells
ANT: adjacent noncancerous tissues
ASK1: apoptosis signal-regulating kinase 1
Gclm: glutamate cysteine ligase
JNK: c-Jun N-terminal kinase
GSH: glutathione
SA: sense-antisense
cis-NATs: cis-natural antisense transcripts
MAPK: mitogen-activated protein kinase
ROC: receiver operating characteristic
ROS: reactive oxygen specie
