# Supplementary material for: Decreased expression of the long non-coding RNA SLC7A11-AS1 predicts poor prognosis and promotes tumor growth in gastric cancer
Source: Oncotarget. 2017 Nov 7;8(68):112530–49. doi: 10.18632/oncotarget.22486 (PMC5762530; doi:10.18632/oncotarget.22486)
Supplement: Supplementary file 1 [file oncotarget-08-112530-s001.pdf]

Decreased expression of the long non-coding RNA SLC7A11-AS1 predicts poor prognosis and promotes tumor growth in gastric cancer

SUPPLEMENTARY MATERIALS

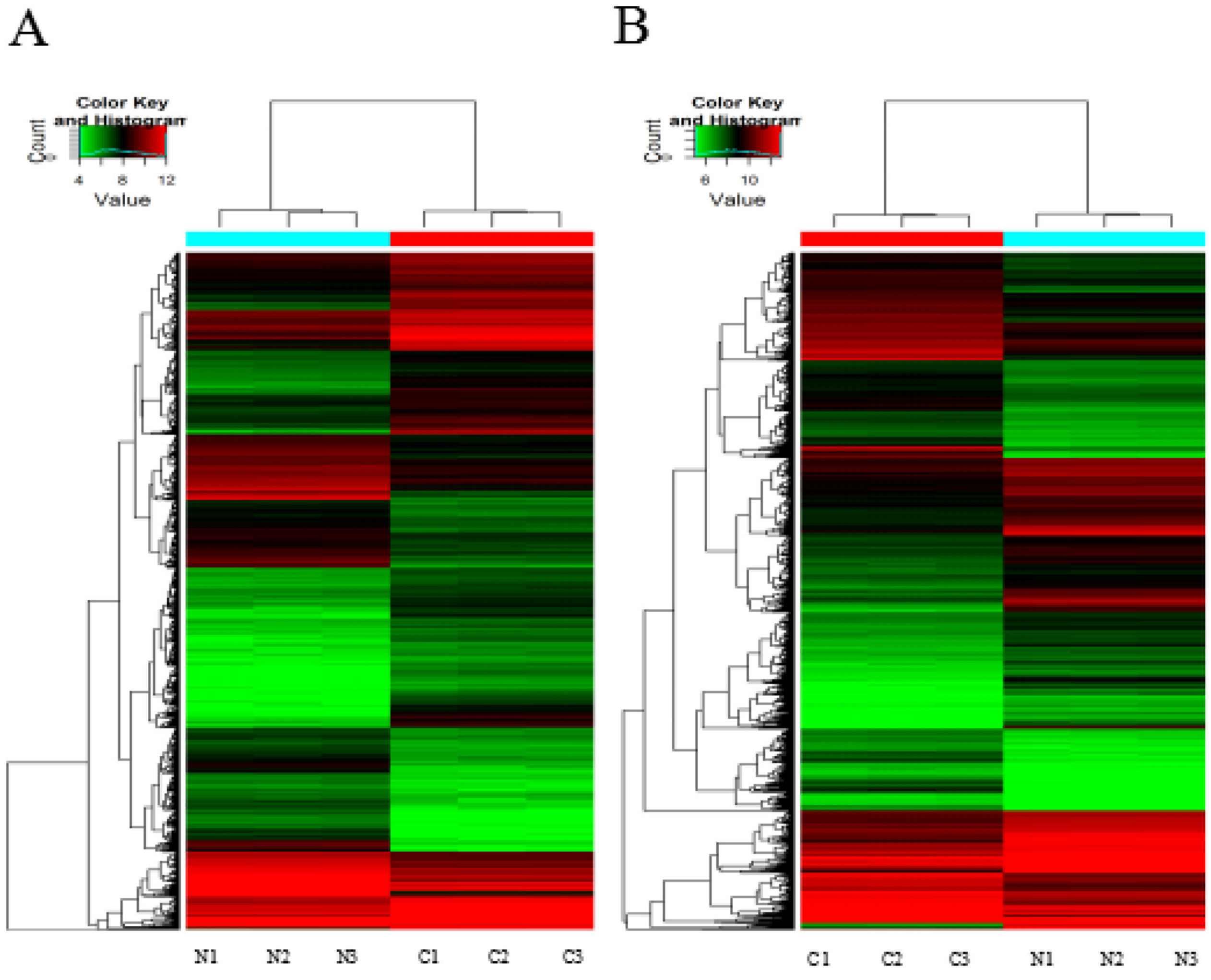

Supplementary Figure 1: Alterations in lncRNA and mRNA expression profiles between 3 paired gastric cancer tissues and paired adjacent noncancerous tissues. (**A**) The result from Hierarchical Clustering heatmap shows distinguishable lncRNA expression profiling among samples. (**B**) The result from Hierarchical Clustering heatmap shows distinguishable mRNA expression profiling among samples. Gene expression levels are indicated as follows: "Red" indicates high relative expression; and "Green" indicates low relative expression.

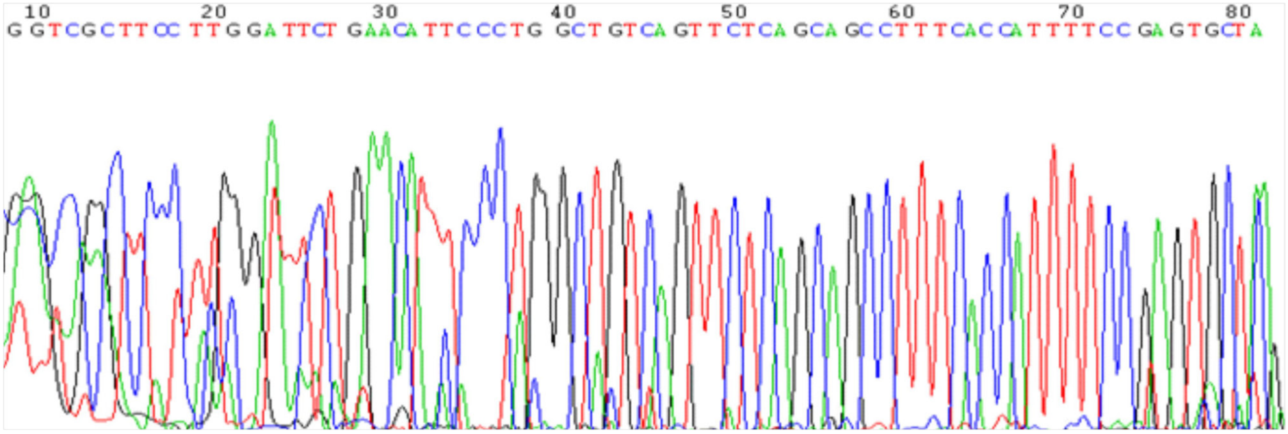

Supplementary Figure 2: Sequencing result of RT-qPCR product of SLC7A11-AS1.

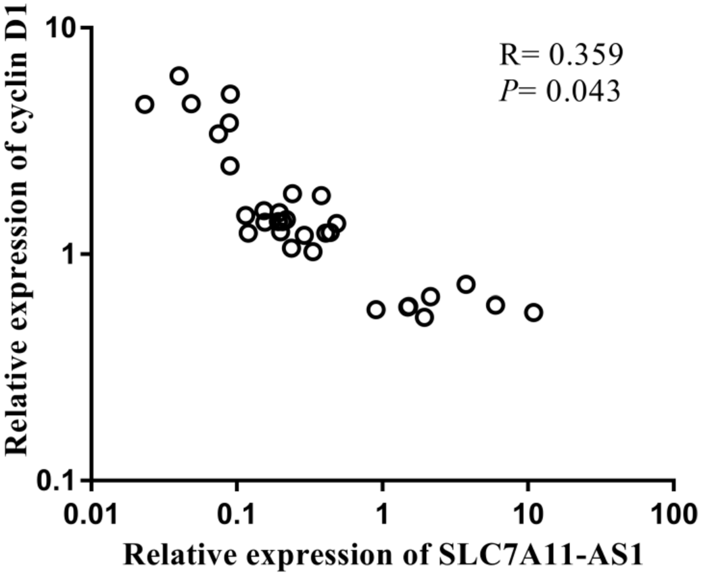

Supplementary Figure 3: The relationship between SLC7A11-AS1 and cyclin D1 expression level. Bivariate correlation analysis of the relationship between SLC7A11-AS1 and cyclin D1 expression level, and the resulting Spearman correlation was calculated as 0.359 where $P = 0.043$ ($N = 32$).

Supplementary Table 1: The primer sequences used for real-time quantitative PCR

Gene name		The primer sequences	Amplification length (bp)
SLC7A11-AS1	Forward	5′-CATCCTGTGGCTGGAGAGAT-3′	110 bp
	Reverse	5′-AGCACTCGGAAAATGGTGAA-3′	
SLC7A11	Forward	5′-ATGCAGTGGCAGTGACCTTT-3′	71 bp
	Reverse	5′-GGCAACAAAGATCGGAACTG-3′	
ASK1	Forward	5′-CCTGAAGCTTAAGTCCCAACC-3′	227 bp
	Reverse	5′-GCATCCCTCCCCTTAGTCTC-3′	
Cyclin D1	Forward	5′-TGGAGGTCTGCGAGGAACA-3′	148 bp
	Reverse	5′-TTCATCTTAGAGGCCACGAACAT-3′	
Gclm	Forward	5′-ACTGACTTAGGAGCATAACTTACC-3′	111 bp
	Reverse	5′-ACTGACTTAGGAGCATAACTTACC-3′	
c-jun	Forward	5′-ATCGACATGGAGTCCCAG-3′	100 bp
	Reverse	5′-ATCGACATGGAGTCCCAG-3′	
p38	Forward	5′-TTCATGCGAAAAGAACCTACAG-3′	138 bp
	Reverse	5′-TGAGATGGGTCACCAGATACAC-3′	
β-actin	Forward	5′-TCCCTGGAGAAGAGCTACGA-3′	194 bp
	Reverse	5′-AGCACTGTGTTGGCGTACAG-3′	
GAPDH	Forward	5′-GCACCGTCAAGGCTGAGAAC-3′	138 bp
	Reverse	5′-TGGTGAAGACGCCAGTGGA-3′	

Supplementary Table 2: SLC7A11-AS1 neighboring genes have disturbed expressions in gastric cancer tissues and paired adjacent noncancerous tissues

Neighbor genes	Fold change	Official full name	Type	Genbank number	*P* value
SLC7A11	10.6365811	cystine/glutamate transporter	coding	NM_014331	0.000314299
LINC00616	1.199951	-	noncoding	NR_037866	0.559868765

Supplementary Table 3: The q-PCR data of relative SLC7A11-AS1 expression level in BGC-823, SGC-7901, MGC-803 and HGC-27 gastric cancer cell lines

BGC-823	SGC-7901*	MGC-803#	HGC-27※
0.838	0.963	1.194	1.511
0.833	1.011	1.298	1.586
0.844	0.963	1.391	1.565

BGC-823 VS SGC-7901, **P* = 0.001; BGC-823 VS MGC-803, #*P* = 0.0013, BGC-823 VS HGC-27, ※*P* < 0.001.

Supplementary Table 4: The q-PCR data of relative SLC7A11-AS1 and SLC7A11 expression levels in SGC-7901, MGC-803 and HGC-27 cells infected adenovirus with sh-RNA by sh-NC and sh-SLC7A11-AS1 vector

Gene	SGC-7901			MGC-803			HGC-27		
	NC	Sh-SLC7A11-AS1	*P*	NC	Sh-SLC7A11-AS1	*P*	NC	Sh-SLC7A11-AS1	*P*
	1.007	0.645		1.049	0.883		0.973	0.933	
SLC7A11-AS1	0.986	0.767	0.001	1.007	0.823	0.023	1.02	0.926	0.008
	1.006	0.663		0.946	0.737		1.007	0.920	
	0.955	1.341		0.920	1.283		0.992	1.372	
SLC7A11	1.059	1.243	0.034	0.966	1.338	0.025	0.993	1.203	0.005
	0.989	1.112		1.125	1.189		0.993	1.254	

Supplementary Table 5: The OD value of cell vitality in SGC-7901, MGC-803 and HGC-27 cells infected adenovirus with sh-RNA by sh-NC and sh-SLC7A11-AS1 vector

Time (hours)	SGC-7901		MGC-803		HGC-27	
	Sh-NC	Sh-SLC7A11-AS1	Sh-NC	Sh-SLC7A11-AS1	Sh-NC	Sh-SLC7A11-AS1
0 h	0.325	0.307	0.364	0.362	0.376	0.438
	0.290	0.300	0.311	0.315	0.416	0.431
	0.279	0.277	0.339	0.337	0.370	0.380
	0.283	0.289	0.313	0.316	0.413	0..394
24 h	0.373	0.461	1.168	1.038	0.689	0.942
	0.370	0.470	1.127	1.096	0.611	0.813
	0.369	0.449	1.138	1.273	0.854	0.864
	0.364	0.459	1.065	1.235	0.869	0.815
48 h	0.619	0.719	1.755	1.785	0.929	1.389
	0.577	0.725	1.884	1.992	1.402	1.572
	0.565	0.754	1.885	2.094	1.414	1.460
	0.550	0.750	1.875	2.093	1.332	1.577
72 h	0.680	0.914	2.506	2.508	2.047	2.562
	0.653	0.919	2.335	2.538	2.274	2.577
	0.651	0.874	2.419	2.575	2.041	2.434
	0.632	0.871	2.395	2.809	2.343	2.547
P value	< 0.001		0.046		< 0.001	

Supplementary Table 6: The data of three phases cell population in SGC-7901, MGC-803 and HGC-27 cells infected adenovirus with sh-RNA by sh-NC and sh-SLC7A11-AS1 vector

Phase	SGC-7901			MGC-803			HGC-27		
	Sh-NC	Sh-SLC7A11-AS1	*P* value	Sh-NC	Sh-SLC7A11-AS1	*P* value	Sh-NC	Sh-SLC7A11-AS1	*P* value
G1	51.96%	36.37%	0.029	61.07%	61.96%	0.621	77.29%	69.66%	0.002
	48.79%	19.20%		59.42%	56.64%		77.25%	67.53%	
	58.41%	37.86%		61.39%	60.55%		80.15%	70.4%	
S	28.79%	48.67%	0.022	33.26%	36.12%	0.013	5.21%	10.29%	0.123
	36.87%	40.64%		33.94%	37.96%		4.35%	10.37%	
	28.12%	44.47%		32.62%	35.74%		6.76%	8.3%	
G2	19.29%	40.16%	0.354	5.67%	1.92%	0.292	17.5%	20.04%	0.051
	14.34%	17.67%		6.64%	1.48%		18.4%	22.1%	
	13.46%	14.96%		5.98%	7.62%		13.09%	21.3%	
